# Maternal Gatekeeping Scale for infancy period (0–24 months) adaptation into Turkish: Mother and Father Form

**DOI:** 10.3389/fpsyg.2024.1474556

**Published:** 2024-10-03

**Authors:** Fatma Elif Ergin, Müberra Demirbaş

**Affiliations:** ^1^Department of Child Development, Ankara Yıldırım Beyazıt University, Ankara, Türkiye; ^2^Department of Child Development, Istanbul Medeniyet University, Istanbul, Türkiye

**Keywords:** maternal gatekeeping, father involvement, father-child relationship, assessment, adaptation

## Abstract

This research aimed to adapt the Maternal Gatekeeping Scale into Turkish for use with infants and to evaluate its validity and reliability with parents. The study employed a descriptive survey model, a quantitative research approach. Participants were selected using criterion sampling, a purposeful sampling technique. The study sample included 305 mothers and 209 fathers with infants aged 0–24 months, who are married, living together, and agreed to participate. The data in the study were collected with the “Demographic Information Form” and “Maternal Gatekeeping Scale” created by the researcher. Confirmatory factor analysis was performed for the construct validity of the Maternal Gatekeeping Scale- Mother Form and Maternal Gatekeeping Scale- Father Form. The internal consistency reliability coefficient of the Maternal Gatekeeping Scale-Mother Form was 0.76; the control sub-dimension was 0.75, the encouragement sub-dimension was 0.81, and the obstacle sub-dimension was 0.76. The internal consistency reliability coefficient of the Maternal Gatekeeping Scale-Father Form was 0.87; the control sub-dimension was 0.83, the encouragement sub-dimension was 0.87, and the obstacle sub-dimension was 0.87. In order to calculate item discriminations, 27% lower-upper groups were formed and independent sample t-test was applied to these groups. Item-total correlation values were calculated to determine the relationship between each item in the scale and other items. The findings of this study demonstrate that both the Mother and Father Forms of the Maternal Gatekeeping Scale are valid and reliable tools for assessing maternal gatekeeping among parents with infants in Türkiye. The adaptation of this scale represents a significant advancement in the field of maternal gatekeeping during infancy. It is anticipated that this adapted scale will serve as a foundational resource for future research, facilitating the exploration of determinants and consequences associated with maternal gatekeeping in infancy.

## 1 Introduction

Infancy, encompassing the first 2 years of life, is a crucial developmental stage that significantly influences lifelong development (Berk, 2013). This period is highly responsive to environmental stimuli, highlighting the importance of caregivers’ roles (Koran, 2016). Notably, the impact on the infant extends beyond the parent-infant relationship; the dynamics between parents also profoundly affect the child (Stocker et al., 1997; Vandewater and Lansford, 1998). Numerous factors influence the father-infant relationship (Dinç and Balcı, 2021), with maternal behavior being a key determinant (De Luccie, 1995). In this context, the concept of maternal gatekeeping, which refers to how maternal behaviors shape the father-child relationship, becomes particularly relevant for study during infancy.

Definitions of maternal gatekeeping have evolved over time, reflecting varying perspectives on its impact. Initially, Allen and Hawkins (1999) defined maternal gatekeeping as “a collection of beliefs and behaviors that ultimately inhibit a collaborative effort between men and women in family work by limiting men’s opportunities for learning and growing through caring for home and children” (Allen and Hawkins, 1999, p. 200). This early definition predominantly emphasized the restrictive and negative aspects of maternal gatekeeping (Puhlman and Pasley, 2013). Conversely, Walker and McGraw (2000) proposed that maternal gatekeeping could act as a facilitator rather than a hindrance (Walker and McGraw, 2000). Building on this perspective, Roy and Dyson (2005) found that fathers viewed their wives’ gatekeeping behaviors as necessary and even encouraging (Roy and Dyson, 2005). Similarly, Sano et al. (2008) argued that mothers used gatekeeping behaviors not to exclude fathers but to guide and regulate their involvement (Sano et al., 2008).

Recent research has aimed to provide a more nuanced understanding of maternal gatekeeping. Puhlman and Pasley (2013) developed a new model incorporating behavioral indicators to capture the complex nature of maternal gatekeeping. Their model, informed by Family Systems Theory and Feminist Theory (Puhlman and Pasley, 2013). Family Systems Theory examines the family as an integrated unit, focusing on the interactions and relationships between its members, as well as its subsystems (Afyonoğlu et al., 2021). Subsystems are interconnected parts of the family system that influence one another. A change in one subsystem impacts the others (Teater, 2015a, pp. 25–32). For instance, the behavior of the mother, a subsystem, affects the father, another subsystem. The mother’s response to the father’s behavior influences how the father reacts to her. Thus, understanding the mother’s actions requires considering the father’s actions, and vice versa. Family Systems Theory also explains how the parenting structure impacts children and how children, in turn, influence the family dynamics (Cox and Paley, 2003; Teater, 2015a, pp. 25–32).

Feminist Theory focuses on the power imbalances and inequalities between women and men (Yeler, 2020, pp. 52–53). It explores how these gender differences and power dynamics affect family roles (Teater, 2015b, pp. 105–121). This theory helps in understanding maternal gatekeeping by highlighting how gender roles and power imbalances within the family impact co-parenting (Allen and Hawkins, 1999).

Examining maternal gatekeeping within the Turkish context is significantly enriched by applying both Family Systems Theory and Feminist Theory. Family Systems Theory provides a framework for understanding how maternal gatekeeping influences family dynamics, given the interconnected nature of family roles in traditional Turkish households. This theory elucidates how maternal behaviors can affect not only the father’s involvement but also the overall functioning of the family system, highlighting the importance of considering these interactions in a culturally specific context. Simultaneously, Feminist Theory offers valuable insights into the role of gender dynamics and societal expectations. In Turkey, where traditional gender roles are prominent, the expectations placed on mothers can shape their approach to parenting and co-parenting. By integrating these theories, the study can explore how cultural norms and power imbalances influence maternal gatekeeping practices, providing a comprehensive understanding of how these factors impact family relationships and dynamics in a specific cultural milieu.

Puhlman and Pasley (2013) defines maternal gatekeeping as “set of complex behavioral interactions between parents, where mothers influence father involvement through their use of controlling, facilitative, and restrictive behaviors directed at father’s childrearing and interaction with children on a regular and consistent basis” (Puhlman and Pasley, 2013, p. 217). To address the complexity of maternal gatekeeping, they proposed a three-dimensional construct comprising “control,” “discouragement,” and “encouragement,” ranging from low to high. The control dimension includes the extent to which the mother is the leader and the final decision maker in family matters and how intensely she supervises the father-child relationship. Mothers with a high level of control have almost all of the decision-making authority in matters related to family and parenting. On the other hand, mothers with low control have little influence over the father and little responsibility in family matters. Maternal control can affect father involvement in both directions; it can both increase and decrease it. The discouragement dimension involves the mother’s setting limits and restricting the father’s relationship with the child and parenting behaviors. Behaviors in the discouragement dimension are seen in the form of criticism, ridicule, and lack of support. Mothers can exhibit their behaviors in the discouragement dimension by explicitly telling fathers or implicitly by implication.The encouragement dimension involves the mother’s support for the father in family and child-related issues. It includes the facilitative and positive effects of maternal gatekeeping on fathers. Behaviors in the encouragement dimension are seen as seeking the father’s opinion on issues related to the child, cooperating with the father, giving importance to rituals related to the father, positive body language and praise.

Before initiating the scale adaptation process, two critical decisions must be addressed: the necessity of adapting the scale and the selection of the most suitable scale for adaptation (Çapık et al., 2018). To determine the necessity, a comprehensive literature review was conducted using keywords such as maternal gatekeeping, father involvement, and co-parenting. The review revealed that maternal behaviors significantly impact the father-child relationship and are characterized as “maternal gatekeeping.” Given the absence of studies on maternal gatekeeping during infancy, it was concluded that research in this area is essential.

Some adaptation studies have been conducted in Türkiye to measure maternal gatekeeping. For example, the mother form of Puhlman and Pasley’s Maternal Gatekeeping Scale (2017) was adapted for mothers with children between the ages of 4 and 6 (Akgöz Aktaş and Aydın, 2020a; Puhlman and Pasley, 2017). The father form of the same scale was adapted for fathers with children between the ages of 3 and 7 (Akgöz Aktaş and Aydın, 2020b). Fagan and Barnett (2003) was adapted for both mothers and fathers with children aged 3–6 years and 7–11 years (Karabulut, 2021). The father form of the same scale was adapted for fathers with an average age of 15.04 years for their children (Karabulut and Şendil, 2017).

Although there are several tools designed to measure gatekeeping among parents of young children, none are specifically tailored for parents of infants in Türkiye. Infancy is the period when the father-child relationship is built (Sarkadi et al., 2008). It is also a critical developmental period in terms of parenting skills as it marks a critical stage in the development of a secure relationship between parents and infant (Haslett and Samter, 2015). Infancy is a critical developmental period where maternal gatekeeping may be particularly pronounced due to the influence of fathers’ involvement on mothers’ self-confidence and perceptions of their maternal identity. This underscores the need for a specialized instrument to accurately capture the dynamics of maternal gatekeeping during this formative stage, thereby addressing a significant gap in the existing research and providing valuable insights into the interplay between parental roles and maternal self-perceptions. In this study, the researchers decided to use the Maternal Gatekeeping Scale (Puhlman and Pasley, 2017), which addresses maternal gatekeeping more comprehensively with different dimensions compared to other scales used in Türkiye and includes the views of mothers and fathers separately.

This study aimed to adapt the Maternal Gatekeeping Scale, developed by Puhlman and Pasley (2013), for use with parents of infants in Türkiye and to evaluate its validity and reliability. By providing a measurement tool specifically designed for this age group, this study is expected to offer valuable data for professionals working with parents of infants. Clinically, this tool can aid in assessing and understanding family dynamics more precisely, enabling practitioners to identify and address issues related to maternal gatekeeping effectively. Additionally, the insights gained from this tool can inform the development of targeted intervention programs aimed at improving parental collaboration and supporting maternal self-perceptions. The originality of this study is further highlighted by the absence of prior research on maternal gatekeeping during infancy in Türkiye. The study’s unique contribution lies in its dual assessment of both mothers’ and fathers’ perspectives, allowing for a comprehensive evaluation of maternal gatekeeping behaviors and perceptions. This approach not only addresses a significant gap in the literature but also facilitates future research on the alignment between mot.

## 2 Materials and methods

### 2.1 Participants

In determining the sample size for the scale adaptation study, it is recommended to have 5–10 times the number of items in the measurement tool to ensure adequate validity and reliability (Field, 2005; Nunnally, 1978). Given that the Maternal Gatekeeping Scale consists of 41 items, the target sample size was set between 205 and 410 participants, in accordance with this guideline.

The study on adapting the Maternal Gatekeeping Scale for infancy included 305 mothers and 209 fathers, all of whom had children aged 0–24 months. Participants were married, living together, and willingly took part in the study. Most of the mothers (47.9%) are between the ages of 26 and 30, while most of the fathers (43.1%) are between the ages of 30–35. Table 1 shows the demographic characteristics of the parents.

**Table 1:** Descriptive statistics of the Mother and Father Forms of the Maternal Gatekeeping Scale.

		Mother Form (*n* = 305)		Father Form (*n* = 209)	
**Variables**		***n***	**%**	***n***	**%**
Age	25 and under	23	7.5	12	5.7
	26–30	146	47.9	60	28.7
	30–35	91	29.8	90	43.1
	35–40	30	9.8	34	16.3
	Over 40 years old	15	4.9	13	6.2
Level of education	Primary school	1	0.3	2	1.0
	Middle school	5	1.6	9	4.3
	High school	30	9.8	31	14.8
	Community college	24	7.9	29	13.9
	Bachelors	169	55.4	88	42.1
	Postgraduate	76	24.9	50	23.9
Diagnosed mental disorder	No	305	100	209	100
	Yes	–	–	–	–
Working status	No	168	55.1	7	3.3
	Yes	137	44.9	202	96.7
Duration of marriage	0–1 year	168	55.1	8	3.8
	1–5 years	90	29.5	98	46.9
	5–10 years	40	13.1	78	37.3
	Over 10 years old	7	2.3	25	12.0
Cohabitation status with spouse	No	–	–	–	–
	Yes	305	100	209	100
Number of children	1	197	64.6	121	57.9
	2	81	26.6	65	31.1
	3	26	8.5	23	11.0
	4	1	0.3	121	57.9
Age of the child	0–6 months	64	21.0	56	26.8
	6–12 months	61	20.0	42	20.1
	12–18 months	65	21.3	39	18.7
	18–24 months	115	37.7	72	34.4

*In table ranges, upper limits are included and lower limits are excluded.

### 2.2 Instruments

The data collection tools used in the study included the “Maternal Gatekeeping Scale- Mother Form,” the “Maternal Gatekeeping Scale- Father Form,” and the “Demographic Information Form” developed by the researcher. The Demographic Information Form comprised questions about parents’ age, education level, presence of any diagnosed mental disorders, employment status, duration of marriage, cohabitation with their spouse, number of children, and the ages of their infants.

The Maternal Gatekeeping Scale was originally developed by Puhlman and Pasley (2017) to be administered to parents with children aged 3–7 years. The scale evaluates mothers’ behaviors towards fathers, specifically in terms of encouragement, control, and discouragement. It consists of 41 items divided into three sub-dimensions and employs a Likert-type format (0-Never, 1-Very Rarely, 2-Rarely, 3-Sometimes, 4-Most of the Time, 5-Always). Higher scores on the subscales indicate greater levels of encouragement, control, or discouragement exhibited by mothers. The Cronbach’s alpha coefficient of the subscales was found to be between 0.74 and 0.94. The Mother Form and Father Form of the Maternal Gatekeeping Scale contain the same questions. Only the subject and predicate conjugations differ according to mothers and fathers.

### 2.3 Procedure

#### 2.3.1 Ethics approval and data collection

Following the approval from the Ethics Committee of authors’ University on May 17, 2023, under decision number 112/05, the scale adaptation process started. Data for the study were collected either face-to-face or online through Google Forms from parents with infants aged 0–24 months.

To qualify for inclusion, participants had to meet the following criteria: (1) be at least 18 years old, (2) have no diagnosed mental health disorders, (3) have a child between 0 and 2 years old, (4) reside with their spouse, (5) be at least literate, and (6) voluntarily agree to participate.

The participants were reached by using the criterion sampling method, which is one of the purposeful sampling methods, in accordance with the criteria above. In the criterion sampling method, the sample consists of people with the characteristics determined in relation to the subject (Büyüköztürk et al., 2008).Within the scope of this study, the inclusion criteria determined in line with the purpose of the research constitute the criteria for sample selection. The mothers and fathers who met the inclusion criteria were reached through the convenient sampling methodology. In this context, the sample of the study consisted of mothers and fathers with infants who agreed to participate in the study throughout Türkiye.

Data collection began with in-person interviews with eligible mothers and fathers in public areas like parks and playgrounds. Participants who consented verbally were asked to sign the Informed Consent Form and then completed the “Demographic Information Form” along with the appropriate Maternal Gatekeeping Scale (Mother or Father Form).

For participants who could not engage in person or were contacted via social media, data were collected via Google Forms. The form included the Informed Consent Form and the Maternal Gatekeeping Scale. Participants received a link through email or social media. They could only access the scale questions after providing consent. The form was designed to direct mothers and fathers to the correct version of the scale based on their responses.

The scale was finalized for data collection and applied to the sample group. After the scale forms were collected, all forms were reviewed and the forms of 16 mothers and 4 fathers who did not meet the inclusion criteria were excluded from the analysis. A total of 514 forms that met the inclusion criteria were included in the analysis.

#### 2.3.2 Scale adaptation process

Erkuş and Selvi (2019) outline a series of stages necessary for adapting a scale (Erkuş and Selvi, 2019). In this study, the adaptation process was conducted through the following steps: (1) securing permission from the original scale’s creator, (2) translating the scale into Turkish, (3) comparing the translation with the original, (4) performing a back translation into the original language, (5) evaluating language equivalence, (6) conducting a pretest, (7) analyzing reliability and validity, and (8) presenting the final version of the adapted scale.

##### 2.3.2.1 Obtaining permission from the researcher who developed the original scale

The scale named “Maternal Gatekeeping Scale” was developed by Puhlman under the supervision of Pasley within the scope of her doctoral dissertation. Puhlman and Pasley were contacted via e-mail. Puhlman returned the e-mail and the necessary permission was obtained for the use of the scale they developed.

##### 2.3.2.2 Translation of the original scale into Turkish

After informing three linguists/translators about the study’s purpose and the scale’s content, the original scale was e-mailed to them. Each linguist independently translated the scale and submitted their translations to the researcher.

##### 2.3.2.3 Comparison of translations

The translations provided by the linguists/translators were reviewed and compared by the researchers. They assessed the translations both conceptually and for their appropriateness in Turkish, subsequently merging them into a single cohesive version.

##### 2.3.2.4 Back translation into the original language

The scale forms combined in a single form were translated from Turkish to English and compared with the items in the original scale. It was seen that the items obtained through back translation were similar to the items in the original scale. Thus, the draft form of the translated scale was formed.

##### 2.3.2.5 Examination of language equivalence

Two approaches were employed to assess language equivalence. The first one was to obtain expert opinion, and the second one was to administer both the English and Turkish versions of the scale to a group fluent in both languages.

##### 2.3.2.6 Expert opinion

Three bilingual experts evaluated the equivalence of the Turkish-translated items against the original English items based on three criteria: (1) Do the Turkish items convey the same meaning as the original English items? (2) Do the words, concepts, and idioms have the same meaning or context in both cultures? (3) Is the language clear and comprehensible? Experts rated each item as “Not Adequate,” “Partially Adequate,” or “Adequate,” and provided suggestions for items rated as “Partially Adequate.” Revisions were made to the scale items based on their feedback.

##### 2.3.2.7 Application to a bilingual group

To evaluate the validity of the translation, both the original English scale and the Turkish-translated scale were administered to a bilingual group. This method involved presenting both versions of the scale in a single session to parents proficient in both languages, which is considered an important approach for assessing translation accuracy (Erkuş and Selvi, 2019). By comparing the scores obtained from both the original and translated scales, statistical analysis was conducted to provide evidence supporting the translation’s adequacy. The correlation analyses for each item, which demonstrate the validity of the translation, are presented in the findings section.

##### 2.3.2.8 Conducting the pretest application

In order to determine whether there were any incomprehensible parts in the scale, the scale was applied to 10 mothers and 10 fathers with infants in the 0–2 age group, and after the application, they were asked whether there were any incomprehensible items. In this way, it was aimed to determine whether the scale had any language and expression problems. No feedback was received from the parents that the items were not comprehensible.

##### 2.3.2.9 Validity and reliability analyses

Validity and reliability analyses of the scale were conducted.

##### 2.3.2.10 Final presentation of the scale

As a result of the steps followed, the final form of the scale was obtained as the Maternal Gatekeeping Scale - Mother Form and Maternal Gatekeeping - Father Form.

### 2.4 Data analysis

SPSS 27 (Statistical Package for the Social Sciences) and LISREL 8.80 programs were used to analyze the data collected in the study. Confirmatory factor analysis was conducted for the construct validity of the Maternal Gatekeeping Scale- Mother Form and Maternal Gatekeeping Scale- Father Form. Frequency, percentage, arithmetic mean and standard deviation were used to determine the distribution of the sample. In terms of validity, we assessed language validity and construct validity. Criterion validity could not be assessed due to the absence of a suitable comparison scale or established gold standard for the instrument. Cronbach’s Alpha, the internal consistency coefficient, was calculated to determine the reliability of the scales used in the study. The correlation coefficient was categorized as follows: very low for values between 0.00 and 0.30, low for values between 0.30 and 0.50, moderate for values between 0.50 and 0.70, high for values between 0.70 and 0.90, and very high for values between 0.90 and 1.00 (Hinkle et al., 2003). To calculate item discriminations, the sample was divided into lower and upper 27% groups. An independent samples t-test was then applied to these groups. Item-total correlation values were calculated to determine the relationship between each item in the scale and other items.

## 3 Results

### 3.1 Language equivalence of the Maternal Gatekeeping Scale- Mother Form

In order to determine the language validity of the Maternal Gatekeeping Scale- Mother Form, the Turkish and English forms of the scale were administered to 31 mothers with a good command of English in the same session (Erkuş and Selvi, 2019). Spearman’s Rho correlation analysis was applied to the items in the Turkish and original forms and the control, encouragement, and discouragement sub-dimensions of the scale. The correlation coefficients obtained from the items and sub-dimensions in the mother form of the scale are given in Table 2.

**Table 2:** Relationships between the items in the Turkish and original forms of the Maternal Gatekeeping Scale - Mother Form.

Turkish Form- original form	*r*	Turkish Form- original form	*r*	Turkish Form- original form	*r*
T1-O1	0.82*	T16-O16	0.77*	T31-O31	0.93*
T2-O2	0.86*	T17-O17	0.70*	T32-O32	0.70*
T3-O3	0.79*	T18-O18	0.82*	T33-O33	0.63*
T4-O4	0.81*	T19-O19	0.96*	T34-O34	0.70*
T5-O5	0.87*	T20-O20	0.68*	T35-O35	0.89*
T6-O6	0.82*	T21-O21	0.79*	T36-O36	0.73*
T7-O7	0.87*	T22-O22	0.86*	T37-O37	0.84*
T8-O8	0.84*	T23-O23	0.86*	T38-O38	0.86*
T9-O9	0.87*	T24-O24	0.73*	T39-O39	0.86*
T10-O10	0.73*	T25-O25	0.77*	T40-O40	0.79*
T11-O11	0.76*	T26-O26	0.97*	T41-O41	0.72*
T12-O12	0.85*	T27-O27	0.82*	Control	0.93*
T13-O13	0.73*	T28-O28	0.73*	Encourage	0.95*
T14-O14	0.85*	T29-O29	0.94*	Discourage	0.90*
T15-O15	0.85*	T30-O30	0.82*	–	–

*p < 0.001.

The Spearman correlation coefficients obtained between the items in Table 2 ranged between 0.63 and 0.97 and were statistically significant (*p* < 0.001). There is a positive, very high and statistically significant (*r* = 0.93; *p* < 0.001) relationship between the control sub-dimensions of the Turkish and English versions of the Maternal Gatekeeping Scale- Mother Form. There is a positive, very high and statistically significant (*r* = 0.95; *p* < 0.001) relationship between the encouragement sub-dimensions of the Turkish and English versions of the Maternal Gatekeeping Scale- Mother Form. There is a positive, very high and statistically significant (*r* = 0.90; *p* < 0.001) correlation between the discouragement sub-dimensions of the Turkish and English versions of the Maternal Gatekeeping Scale- Mother Form.

### 3.2 Language equivalence of the Maternal Gatekeeping Scale- Father Form

The Spearman correlation coefficients obtained between the items in Table 3 ranged between 0.68 and 1 and were statistically significant (*p* < 0.001).

**Table 3:** Relationships between the items in the Turkish and original forms of the Maternal Gatekeeping Scale - Father Form.

Turkish form- original form	*r*	Turkish form- original form	*r*	Turkish form- original form	*r*
T1-O1	0.91*	T16-O16	0.94*	T31-O31	0.80*
T2-O2	0.70*	T17-O17	0.97*	T32-O32	0.95*
T3-O3	0.93*	T18-O18	0.91*	T33-O33	0.82*
T4-O4	0.75*	T19-O19	0.95*	T34-O34	0.95*
T5-O5	0.99*	T20-O20	0.96*	T35-O35	0.90*
T6-O6	0.94*	T21-O21	1.00*	T36-O36	0.91*
T7-O7	0.99*	T22-O22	1.00*	T37-O37	0.92*
T8-O8	0.98*	T23-O23	0.74*	T38-O38	0.94*
T9-O9	0.80*	T24-O24	0.71*	T39-O39	0.94*
T10-O10	0.86*	T25-O25	0.99*	T40-O40	0.68*
T11-O11	0.94*	T26-O26	0.99*	T41-O41	0.98*
T12-O12	0.97*	T27-O27	0.96*	Control	0.86*
T13-O13	0.95*	T28-O28	0.70*	Encourage	0.99*
T14-O14	0.97*	T29-O29	0.85*	Discourage	0.97*
T15-O15	0.76*	T30-O30	0.89*	–	–

*p < 0.001.

There is a positive, high and statistically significant (*r* = 0.86; *p* < 0.001) relationship between the control sub-dimensions of the Turkish and English versions of the Maternal Gatekeeping Scale- Father Form. There is a positive, very high and statistically significant (*r* = 0.99; *p* < 0.001) relationship between the encouragement sub-dimensions of the Turkish and English versions of the Maternal Gatekeeping Scale- Father Form. There is a positive, very high and statistically significant (*r* = 0.97; *p* < 0.001) correlation between the discouragement sub-dimensions of the Turkish and English versions of the Maternal Gatekeeping Scale- Father Form.

### 3.3 Construct validity

Confirmatory factor analysis was performed to validate the construct of the Maternal Gatekeeping Scale.

The scale, consisting of 41 items, was analyzed with the following item groupings: items 2, 6, 7, 10, 13, 21, 22, 27, 30, 32, 33 as indicators of the control sub-dimension; items 3, 8, 9, 11, 14, 17, 18, 23, 24, 26, 28, 31, 35, 39 as indicators of the encouragement sub-dimension; and items 1, 4, 5, 12, 15, 16, 19, 20, 25, 29, 34, 36, 37, 38, 40, 41 as indicators of the discouragement sub-dimension. Prior to model testing, item 7 was recoded to ensure its consistency in meaning with the other scale items. CFA analysis was conducted using the LISREL package program. In the literature, in evaluating the model fit of a measurement tool: Chi-square (X2) Goodness of Fit, Goodness of Fit Index (GFI), Adjusted Fit Index (AGFI), Root Mean Square Error of Approximation (RMSEA), Square Root of Standardized Residual Means (SRMR), Comparative Fit Index (CFI), Normed Fit Index (NFI) and Non-Normed Fit Index (NNFI) (Çokluk et al., 2012). In this direction, considering the goodness of fit values in Table 4, the models established for the Mother and Father Forms as a result of the confirmatory factor analysis obtained in structural equation modeling were evaluated (Çelik and Yılmaz, 2013).

**Table 4:** Goodness of fit values for confirmatory factor analysis model.

Compliance measure	Good compliance	Acceptable compliance	Mother form	Father form
χ ^2^/sd	0≤ χ ^2^/sd ≤ 2	2≤ χ ^2^/sd ≤ 5	1784.06/776 = 2.30	1962.60/776 = 2.53
RMSEA	0 ≤ RMSEA≤0.05	0.05 ≤ RMSEA≤0.08	0.074	0.086
SRMR	0 ≤ SRMR≤0.05	0.05 ≤ SRMR≤0.08	0.082	0.092
NFI	0.95 ≤ NFI < 1	0.90 ≤ NFI < 0.95	0.85	0.88
CFI	0.95 ≤ CFI < 1	0.90 ≤ CFI < 0.95	0.91	0.93
GFI	0.95 ≤ GFI < 1	0.90 ≤ GFI < 0.95	0.75	0.68

When Table 4 is examined, while chi-square value/degree of freedom, RMSEA and CFI values of the mother form model obtained by confirmatory factor analysis show acceptable fit (*χ*^2^/sd ≤ 5; RMSEA≤0.08; CFI ≥ 0.90); SRMR, NFI and GFI values are not within the desired range (SRMR≥0.08; NFI ≤ 0.90; GFI ≤ 0.90). Since half of the goodness-of-fit values are within the desired range, it can be said that the Maternal Gatekeeping Scale - Mother Form provides model-data fit.

**Figure 1:**
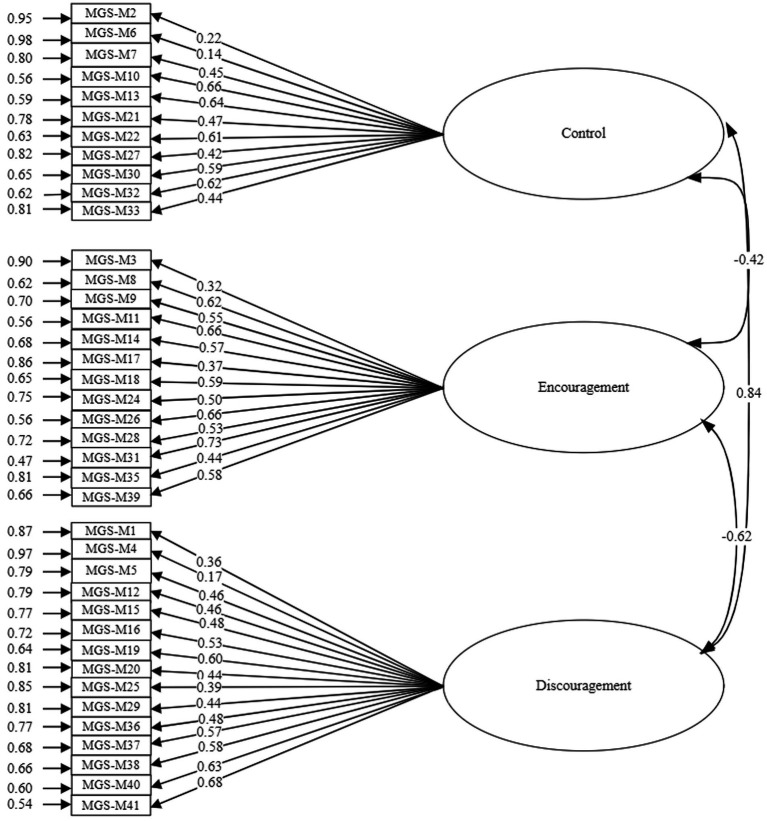
Standardized analytic values of the model obtained by confirmatory factor analysis of the Maternal Gatekeeping Scale Mother Form.

Figure 1 shows the model with the standardized analysis values of the structure obtained as a result of the confirmatory factor analysis of the Mother Form.

When Table 4 is examined, while the chi-square value/degree of freedom and CFI values of the Father Form model obtained by confirmatory factor analysis show acceptable fit (*χ*^2^/sd ≤ 5; CFI ≥ 0.90); RMSEA, SRMR, NFI and GFI values are not within the desired range (RMSEA≥0.08; SRMR≥0.08; NFI ≤ 0.90; GFI ≤ 0.90). RMSEA values between 0.08 and 1 are also considered as poor fit (Fabrigar et al., 1999). Since half of the goodness-of-fit values were within the desired range, it can be said that the paternal form of the Maternal Gatekeeping Scale provided model-data fit.

**Figure 2:**
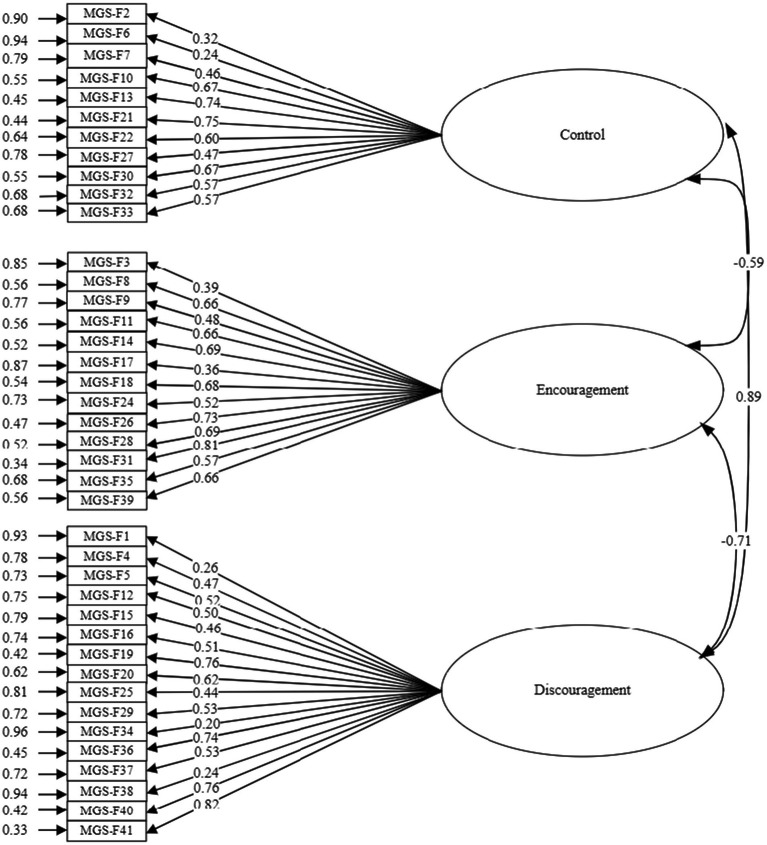
Standardized analytic values of the model obtained by confirmatory factor analysis of the Maternal Gatekeeping Scale Father Form.

Figure 2 shows the model with the standardized analysis values of the structure obtained as a result of the confirmatory factor analysis of the Father Form.

In the Maternal Gatekeeping Scale - Mother Form, items 23 and 34 were classified under different latent variables. Specifically, item 23 (*t* = 1.72; *p* > 0.05) and item 34 (*t* = 1.62; *p* > 0.05) did not show statistically significant predictions by the encouragement and obstacle latent variables, respectively. Consequently, these two items were excluded from the Mother Form, and the model was reanalyzed. Table 5 presents the factor loadings, standard errors, and t-values from the confirmatory factor analysis of the revised 39-item Mother Form.

**Table 5:** Parameter values of the model obtained by confirmatory factor analysis of Maternal Gatekeeping Scale - Mother Form.

Latent and observed variables	Standard error	Standardized factor loadings	*t*-values	*R* ^2^
**Control**				
I2	0.09	0.22	3.75	0.05
I6	0.09	0.14	2.12	0.02
I7	0.07	0.45	7.69	0.20
I10	0.06	0.66	12.08	0.43
I13	0.05	0.64	11.64	0.41
I21	0.06	0.47	8.05	0.22
I22	0.08	0.61	10.95	0.37
I27	0.09	0.42	7.19	0.18
I30	0.06	0.59	10.52	0.35
I32	0.06	0.62	11.33	0.39
I33	0.09	0.44	7.38	0.19
**Encourage**				
I3	0.09	0.32	5.37	0.10
I8	0.06	0.62	11.15	0.38
I9	0.06	0.55	9.77	0.30
I11	0.07	0.66	12.27	0.44
I14	0.05	0.57	10.32	0.33
I17	0.07	0.37	6.32	0.14
I18	0.06	0.59	10.64	0.35
I24	0.08	0.50	8.83	0.25
I26	0.07	0.66	12.11	0.43
I28	0.06	0.53	9.37	0.28
I31	0.05	0.73	13.95	0.53
I35	0.08	0.44	7.60	0.19
I39	0.05	0.58	10.43	0.34
**Discourage**				
I1	0.09	0.36	6.18	0.13
I4	0.10	0.17	2.94	0.03
I5	0.07	0.46	7.91	0.21
I12	0.08	0.46	7.99	0.21
I15	0.08	0.48	8.52	0.23
I16	0.08	0.53	9.39	0.28
I19	0.04	0.60	11.03	0.36
I20	0.08	0.44	7.44	0.19
I25	0.08	0.39	6.50	0.15
I29	0.07	0.44	7.59	0.19
I36	0.04	0.48	8.36	0.23
I37	0.07	0.57	10.43	0.33
I38	0.03	0.58	10.56	0.34
I40	0.04	0.63	11.72	0.40
I41	0.03	0.68	12.74	0.46

Upon reviewing Table 5, which details the parameters from the confirmatory factor analysis of the 39-item Maternal Gatekeeping Scale- Mother Form, it is observed that the standardized factor loadings range from 0.14 to 0.73. All factor loadings listed are statistically significant (*t* > 1.96; *p* < 0.05), indicating that the observed variables are significant predictors of the latent variables.

Table 6 presents the model-data fit indices for the 39-item Maternal Gatekeeping Scale- Mother Form. According to the table, the chi-square value/degree of freedom, RMSEA, and CFI values indicate an acceptable fit for the model (*χ*^2^/df ≤ 5; RMSEA ≤0.08; CFI ≥ 0.90). However, the SRMR, NFI, and GFI values fall outside the desired range (SRMR ≥0.08; NFI ≤ 0.90; GFI ≤ 0.90). Since half of the goodness-of-fit values are within the desired range, it can be said that the 39-item Maternal Gatekeeping Scale-Mother Form provides model-data fit.

**Table 6:** Goodness of fit values for the confirmatory factor analysis model of the Maternal Gatekeeping Scale-Mother Form.

Model	χ ^2^/sd	RMSEA	SRMR	NFI	CFI	GFI
39-Items Mother Form	1935.53/699 = 2.77	0.76	0.081	0.86	0.91	0.75

Table 7 illustrates the relationships between the latent variables in the Maternal Gatekeeping Scale- Mother Form. According to the table, there is a negative, low, and statistically significant relationship between the control latent variable and the encouragement latent variable (*r* = −0.42; *p* < 0.001). There is a positive, high and statistically significant (*r* = −0.84; *p* < 0.001) relationship between the control latent variable and the discouragement latent variable of the Maternal Gatekeeping Scale- Mother Form. There is a negative, moderate and statistically significant (*r* = −0.62; *p* < 0.001) relationship between the encouragement latent variable and the discouragement latent variable of the Maternal Gatekeeping Scale- Mother Form.

**Table 7:** Relationships between latent variables in the model obtained by confirmatory factor analysis of the Maternal Gatekeeping Scale - Mother Form.

Latent variables	1	2	3
Control	–		
Encourage	−0.42*	–	
Discourage	0.84*	−0.62*	–

*p < 0.001.

Item 23 (*t* = 0.53; *p* > 0.05) in the Maternal Gatekeeping Scale- Father Form did not show a statistically significant prediction by the encouragement latent variable. Consequently, this item was removed from the scale, and the model was reanalyzed. Table 8 presents the factor loadings, standard errors, and t-values resulting from the confirmatory factor analysis of the revised 40-item Father Form.

**Table 8:** Parameter Values of the model obtained by confirmatory factor analysis of the Maternal Gatekeeping Scale - Father Form.

Latent and observed variables	Standard error	Standardized factor loadings	*t*-values	*R* ^2^
**Control**				
I2	0.11	0.32	4.47	0.10
I6	0.12	0.24	3.41	0.06
I7	0.09	0.46	6.59	0.21
I10	0.08	0.67	10.48	0.45
I13	0.07	0.74	12.02	0.55
I21	0.08	0.75	12.16	0.56
I22	0.10	0.60	9.07	0.36
I27	0.11	0.47	6.83	0.22
I30	0.09	0.67	10.51	0.45
I32	0.10	0.57	8.48	0.32
I33	0.10	0.57	8.70	0.33
**Encourage**				
I3	0.10	0.39	5.52	0.15
I8	0.08	0.66	10.41	0.44
I9	0.07	0.48	6.99	0.23
I11	0.09	0.66	10.40	0.44
I14	0.08	0.69	10.86	0.47
I17	0.12	0.36	5.09	0.13
I18	0.09	0.68	10.76	0.46
I24	0.10	0.52	7.73	0.27
I26	0.09	0.73	11.77	0.53
I28	0.07	0.69	11.01	0.48
I31	0.07	0.81	13.91	0.66
I35	0.10	0.57	8.75	0.33
I39	0.07	0.66	10.34	0.43
**Discourage**				
I1	0.11	0.26	3.67	0.07
I4	0.10	0.47	6.92	0.22
I5	0.08	0.52	7.90	0.27
I12	0.10	0.50	7.53	0.25
I15	0.10	0.46	6.74	0.21
I16	0.10	0.51	7.73	0.26
I19	0.06	0.76	12.78	0.58
I20	0.09	0.62	9.59	0.38
I25	0.09	0.44	6.42	0.19
I29	0.10	0.53	8.02	0.28
I34	0.12	0.20	2.75	0.04
I35	0.10	0.57	8.75	0.33
I36	0.07	0.74	12.30	0.55
I37	0.09	0.53	7.98	0.28
I38	0.06	0.24	13.16	0.06
I40	0.08	0.76	12.74	0.58
I41	0.7	0.82	14.24	0.67

Upon reviewing Table 8, it is observed that the standardized factor loadings for the 40-item Maternal Gatekeeping Scale- Father Form range from 0.20 to 0.82. Since all factor loadings in the table are statistically significant (t > 1.96; *p* < 0.05), it can be said that observed variables are significant predictors of latent variables.

The 40-item Maternal Gatekeeping Scale-Father Form model-data fit indices are given in Table 9. When Table 9 is examined, while chi-square value/degree of freedom and CFI values of the Father Form model obtained by confirmatory factor analysis show acceptable fit (χ2/sd ≤ 5; CFI ≥ 0.90); RMSEA shows poor fit (0.08 ≤ RMSEA≤1); SRMR, NFI and GFI values are not within the desired range (SRMR≥0.08; NFI ≤ 0.90; GFI ≤ 0.90). Since half of the goodness-of-fit values are within the desired range, it can be said that the 40-item Maternal Gatekeeping Scale- Father form provides model-data fit.

**Table 9:** Goodness of fit values for confirmatory factor analysis model of Maternal Gatekeeping Scale - Father Form.

Model	χ ^2^/sd	RMSEA	SRMR	NFI	CFI	GFI
40-Items Father Form	1.654,05/737 = 2.44	0.08	0.089	0.89	0.93	0.70

Table 10 illustrates the relationships between the latent variables in the Maternal Gatekeeping Scale- Father Form. Analysis of Table 10 reveals a negative, moderate, and statistically significant correlation (*r* = −0.59; *p* < 0.001) between the control and encouragement latent variables in the scale. There is a positive, high and statistically significant (*r* = 0.89; *p* < 0.001) relationship between the control latent variable and the discouragement latent variable of the Maternal Gatekeeping Scale- Father Form. There is a negative, high and statistically significant (*r* = −0.71; *p* < 0.001) relationship between the encouragement latent variable and the discouragement latent variable.

**Table 10:** Relationships between latent variables in the model obtained by confirmatory factor analysis of the Maternal Gatekeeping Scale - Mother Form.

Latent variables	1	2	3
Control	–		
Encourage	−0.59*	–	
Discourage	0.89*	−0.71*	–

*p < 0.001.

### 3.4 Internal consistency reliability

Cronbach’s Alpha was calculated to calculate the internal consistency reliability of the 39-item Maternal Gatekeeping Scale- Mother Form. The internal consistency reliability coefficient for the Mother Form of the scale was 0.76; the control sub-dimension was 0.75, the encouragement sub-dimension was 0.81, and the obstacle sub-dimension was 0.76.

Cronbach’s Alpha was calculated to evaluate the internal consistency reliability of the 40-item Maternal Gatekeeping Scale- Father Form. The overall internal consistency reliability coefficient for the Father Form was 0.87. Specifically, the reliability coefficients for the sub-dimensions were as follows: control sub-dimension, 0.83; encouragement sub-dimension, 0.87; and obstacle sub-dimension, 0.87.

### 3.5 Item statistics

#### 3.5.1 Item statistics for the Mother Form

In order to determine the discrimination of the items of the Maternal Gatekeeping Scale- Mother Form, the upper and lower 27% groups were determined and the total scores of these groups were compared. In addition, item-total correlations were calculated and the relationship between the item and the total score obtained from other items was determined. The analysis results of the mother form are presented in Table 11.

**Table 11:** Item statistics of the Maternal Gatekeeping Scale - Mother Form.

Items	$\bar{X}$	*ss*	*t*	*r* (Corrected item total correlation)	Cronbach’s *α* (scale reliability coefficient after item removal)
I1	1.46	1.45	−6.52**	0.37	0.84
I2	2.42	1.49	−7.09**	0.42	0.84
I3	3.67	1.51	−6.01**	0.41	0.84
I4	1.12	1.74	−1.12	0.07	0.85
I5	1.13	1.17	−5.87**	0.36	0.84
I6	3.13	1.51	−7.46**	0.48	0.84
I7	1.41	1.33	−5.30**	0.29	0.85
I8	4.12	1.17	−5.30**	0.10	0.85
I9	4.30	1.18	−1.67	0.11	0.85
I10	0.88	1.19	−1.91	0.55	0.84
I11	3.77	1.39	−7.83**	0.08	0.85
I12	1.66	1.45	−1.17	0.47	0.84
I13	0.61	1.05	−7.02**	0.40	0.84
I14	4.43	0.95	−5.27**	−0.01	0.85
I15	1.53	1.52	0.24	0.44	0.84
I16	1.30	1.51	−5.80**	0.46	0.84
I17	3.98	1.34	−6.77**	0.28	0.85
I18	4.08	1.22	−3.60**	0.15	0.85
I19	0.32	0.91	−1.47	0.32	0.85
I20	0.95	1.39	−3.72**	0.42	0.84
I21	0.68	1.21	−6.57**	0.41	0.84
I22	1.31	1.52	−4.98**	0.60	0.84
I24	3.05	1.61	−9.63**	0.37	0.84
I25	1.05	1.35	−5.37**	0.46	0.84
I26	3.99	1.30	−6.24**	0.13	0.85
I27	2.40	1.54	−1.25	0.49	0.84
I28	4.27	1.12	−8.24**	0.20	0.85
I29	0.74	1.25	−2.54*	0.41	0.84
I30	0.73	1.16	−5.38**	0.44	0.84
I31	4.51	0.96	−6.19**	−0.02	0.85
I32	0.73	1.24	0.00	0.60	0.84
I33	1.89	1.71	−8.07**	0.59	0.84
I35	4.11	1.47	−11.52**	0.14	0.85
I36	0.31	0.86	−2.46*	0.32	0.85
I37	0.88	1.40	−3.55**	0.31	0.85
I38	0.14	0.60	−3.97**	0.30	0.85
I39	4.60	1.01	−2.22*	0.04	0.85
I40	0.35	0.92	−1.00	0.32	0.85
I41	0.21	0.68	−3.58**	0.38	0.85

*p < 0.05; **p < 0.001.

According to Table 11, in the analyses based on the difference between the lower and upper groups according to the Maternal Gatekeeping Scale- Mother Form, a statistically significant difference was found in items other than items 4, 9, 10, 12, 15, 19, 27, 32 and 40 (*p* < 0.05). This indicates that the scale effectively differentiates between the upper and lower groups, with most items demonstrating significant discriminatory power. Despite the non-significance of these specific items in the group comparison, they were retained in the scale due to their statistically significant factor loadings in confirmatory factor analysis. The item-total correlation values, ranging from 0.01 to 0.60, were considered acceptable for retaining items with correlations below 0.30, as they still contributed to item discrimination in the group analysis. Additionally, the Cronbach’s Alpha values remained consistent when these items were removed, supporting their discriminative validity.

#### 3.5.2 Item statistics for the Father Form

In order to determine the discrimination of the items of the Maternal Gatekeeping Scale-Father Form, a 27% lower-upper group was determined in the same way as the Mother Form and the total scores of these groups were compared. In addition, item-total correlations were calculated and the relationship between the item and the total score obtained from other items was determined.

Table 12 presents the analysis results for the Maternal Gatekeeping Scale- Father Form. The data reveal a statistically significant difference between the lower and upper groups for all items except items 8, 9, 11, 14, 18, 26, 31, 35, and 39 (*p* < 0.05). This indicates that the scale generally exhibits strong discriminatory power across the items. Despite the non-significance of these particular items in the group comparison, they were retained due to their significant factor loadings in confirmatory factor analysis. The item-total correlation values range from 0.01 to 0.70. Items with correlations above 0.30 are considered discriminative (Kline, 2023), while those below this threshold were still included in the scale because they contributed to item discrimination in the group comparison analysis. Furthermore, the Cronbach’s Alpha remained unchanged when these items were removed, confirming their discriminative validity.

**Table 12:** Item statistics of the Maternal Gatekeeping Scale - Father Form.

Items	$\bar{X}$	ss	*t*	*r* (Corrected item total correlation)	Cronbach’s *α* (scale reliability coefficient after item removal)
I1	1.56	1.70	−6.02***	0.45	0.87
I2	2.66	1.64	−7.42***	0.57	0.87
I3	3.57	1.63	−5.44***	0.44	0.87
I4	0.97	1.49	−4.41***	0.30	0.87
I5	1.08	1.32	−4.89***	0.40	0.87
I6	2.57	1.76	−7.16***	0.49	0.87
I7	1.25	1.42	−2.47*	0.18	0.87
I8	3.77	1.35	0.26	0.01	0.88
I9	4.26	0.99	−0.91	0.10	0.87
I10	1.26	1.45	−6.67***	0.57	0.87
I11	3.37	1.47	−0.17	−0.01	0.88
I12	1.71	1.65	−8.24***	0.61	0.86
I13	1.02	1.40	−5.93***	0.50	0.87
I14	3.90	1.38	0.60	−0.10	0.88
I15	1.85	1.67	−5.30***	0.43	0.87
I16	1.37	1.55	−6.64***	0.48	0.87
I17	3.28	1.76	−2.93**	0.25	0.87
I18	3.68	1.51	0.16	−0.09	0.88
I19	0.68	1.23	−4.79***	0.47	0.87
I20	1.25	1.67	−7.19***	0.53	0.87
I21	0.89	1.46	−6.13***	0.59	0.87
I22	1.66	1.69	−8.63***	0.63	0.86
I24	3.08	1.67	−3.18**	0.28	0.87
I25	1.30	1.43	−6.33***	0.56	0.87
I26	3.44	1.66	1.10	−0.16	0.88
I27	2.54	1.74	−7.15***	0.51	0.87
I28	4.02	1.31	−0.99	0.14	0.87
I29	1.51	1.69	−5.98***	0.50	0.87
I30	1.15	1.53	−8.76***	0.69	0.86
I31	3.93	1.36	0.41	−0.07	0.88
I32	1.35	1.70	−7.08***	0.56	0.87
I33	1.92	1.69	−11.04***	0.70	0.86
I34	1.85	1.76	−4.54***	0.32	0.87
I35	3.75	1.59	−0.78	0.03	0.88
I36	0.58	1.18	−5.56***	0.57	0.87
I37	1.21	1.55	−5.92***	0.50	0.87
I38	0.58	1.20	−4.62***	0.51	0.87
I39	4.30	1.11	1.90	−0.18	0.88
I40	0.91	1.52	−3.93***	0.41	0.87
I41	0.76	1.38	−5.70***	0.52	0.87

*p < 0.05; **p < 0.01; ***p < 0.001.

## 4 Discussion

In this study, the Maternal Gatekeeping Scale was adapted into Turkish to be used for assessing maternal gatekeeping during infancy. A comprehensive validity and reliability study was conducted with parents, resulting in the development of the Maternal Gatekeeping Scale-Mother Form and Maternal Gatekeeping Scale- Father Form for parents of infants aged 0–24 months. The findings indicate that the Maternal Gatekeeping Scale is a valid and reliable tool for measuring maternal gatekeeping among mothers and fathers in Türkiye, based on data collected from married and cohabiting parents.

### 4.1 Language equivalence

In order to determine the linguistic validity of the Maternal Gatekeeping Scale- Mother Form, the Turkish and English forms of the scale were administered to 31 mothers with a good command of English in the same session. As a result of the statistical analysis, a positive, very high and statistically significant relationship was found in all sub-dimensions. In order to determine the language validity of the Maternal Gatekeeping Scale- Father Form, the Turkish and English versions of the scale were administered to 20 fathers who were fluent in English in the same session. In previous studies that adapted the Maternal Gatekeeping Scale to different age groups and contexts, including adaptations within Turkey, there was no analysis of linguistic equivalence (Akgöz Aktaş and Aydın, 2020a; Akgöz Aktaş and Aydın, 2020b; Karabulut and Şendil, 2017). Additionally, the Japanese adaptation of the scale did not include a bilingual group for comparison (Kaneko and Hamaguchi, 2020). Sireci and Berberoglu (2000) have highlighted that merely translating and comparing scales is insufficient for ensuring linguistic equivalence. To address this gap, our study included an analysis of language equivalence by calculating the correlation coefficient between the English (original) and Turkish versions of the scale. This analysis involved a small group of bilingual individuals and demonstrated a high correlation coefficient, thereby supporting the linguistic equivalence of the translated scale and minimizing language-related issues.

### 4.2 Construct validity

Confirmatory factor analysis (CFA) was conducted to ensure the construct validity of the Maternal Gatekeeping Scale. CFA is a multivariate analysis that tests whether a pre-existing, defined and restricted structure is confirmed as a model (Çokluk et al., 2012; Keith and Reynolds, 2018). In scale adaptation studies, CFA is often preferred due to its ability to test predefined sub-dimensions and factor structures of an existing scale (Fabrigar et al., 1999; Watkins, 1989). Given that the Maternal Gatekeeping Scale has established sub-dimensions and a confirmed factor structure, CFA was employed to assess its construct validity. This method is particularly effective in evaluating the validity of the scale’s factor structure, aligning with its prior confirmation and enhancing the robustness of the adaptation process. When the scale adaptation studies in the Turkish literature are examined, it is seen that the construct validity was investigated with CFA (Karakuş et al., 2016; Özcan and Koca, 2019). As a result of CFA, it was determined that the three sub-dimensional structure of the Mother Form of the scale, similar to the original, was confirmed with 39 items for the current sample, while the Father Form was confirmed with 40 items. The fact that the standard loadings and loadings were significant, and the fit indices supported the model showed that the Turkish adaptation of the three-dimensional structure of the Maternal Gatekeeping Scale can be valid for parents with infants aged 0–24 months.

When interpreting the results, it is important to consider the characteristics of the sample. This study’s sample consists of relatively young, newly married individuals, with many participants having only one child. Such characteristics can influence the goodness-of-fit indices differently. Specifically, RMSEA is less sensitive to sample size compared to NFI and GFI (Kline, 2023; Rigdon, 1996). Consequently, a smaller sample size may result in lower NFI and GFI values. Indeed, the larger sample size in the Mother Form has contributed to better goodness-of-fit values compared to the Father Form.

In the adaptation of the Maternal Gatekeeping Scale, certain items were excluded based on their performance across different forms. Specifically, item 23 “I leave home so that he can take care of the child” in the Mother Form and “She leaves the house so that I can take care of the child” in the Father Form was excluded from both forms. This item had a t-value below 1.96 (*p* > 0.05), indicating it was not significantly predicted by the latent variables. The failure of this item in both forms may reflect cultural differences influencing how these roles and behaviors are perceived and reported. Especially in Turkey, the defined roles of men and women and the mother’s primary role in childcare and development (Sunar and Fişek, 2005) may be the reason. Similarly item 34 “She acts as if he supports my decisions about parenting (even if he does not)” was found to be non-operational in the Mother Form. This discrepancy may stem from the nature of self-reporting biases, where mothers might rate their partners’ support differently from how fathers perceive it. The literature supports that different items can be functional in Mother and Father Forms of scales, highlighting varying perceptions of gatekeeping behaviors (Akgöz Aktaş and Aydın, 2020a; Akgöz Aktaş and Aydın, 2020b; Puhlman and Pasley, 2013; Sucuoğlu et al., 2015).The discrepancy between items in Mother and Father Forms may be attributed to the subjective nature of self-reports and the differing perspectives of the influencer (mother) and the influenced (father) (Fagan and Barnett, 2003; Puhlman and Pasley, 2017).

It was found that all of the relationships between the sub-dimensions were significant in both the Mother and Father Forms. Although the relationship between the barrier and encouragement dimensions was at a moderate level for mothers, it was higher for fathers. Although the control and encouragement dimensions were at a low level for mothers, they were at a medium level for fathers. Puhlman and Pasley (2017) states that this can be explained by the fact that men’s thoughts are more dichotomous (Puhlman and Pasley, 2017). In the original study, similar to the findings of this study, it was reported that there was a significant negative relationship between the encouragement and discouragement dimensions of the scale, and a significant positive relationship between the discouragement and control dimensions. However, in adaptation studies of the Maternal Gatekeeping Scale, different patterns have emerged across various contexts. For instance, research involving different age groups reported a negative relationship between encouragement and discouragement dimensions, a positive relationship between discouragement and control dimensions, and a negative relationship between encouragement and control dimensions (Akgöz Aktaş and Aydın, 2020a; Kıraçcı, 2021). Conversely, an adaptation study focusing on parents of children with special needs found a negative, though not significant, relationship between encouragement and control dimensions. This study also observed a significant negative correlation between encouragement and discouragement, as well as a significant positive correlation between discouragement and control dimensions (Kıraçcı, 2021). In the Japanese adaptation study of the scale, a positive correlation was found between the control and encouragement sub-dimensions. Similarly, a positive correlation was found between the control and discouragement dimensions (Kaneko and Hamaguchi, 2020). These findings suggest that while some relationships are consistent, the nature and significance of these relationships can vary depending on the specific population studied and culture.

This study was conducted with married and cohabiting parents with infants aged 0–24 months. It can be tested in other sample groups in future studies. For example, validity and reliability studies can be conducted with parents in different family structures such as divorced parents, foster parents, stepparents, and families with infants with special needs. Using the Maternal Gatekeeping-Mother Form and Father Form, mothers’ evaluations of their own gatekeeping behaviors and fathers’ perceived maternal gatekeeping behaviors can be compared. Clinical and intervention programs can be designed and implemented to increase father involvement and co-parenting by evaluating the impact of mothers on fathers through the scale.

### 4.3 Limitations

While this research makes significant contributions, it also has some limitations. The data are limited to married mothers and fathers with infants between 0 and 24 months, so the validity of the scales for different family types, such as divorced parents, foster parents, or stepparents, remains unknown. Moreover, due to time constraints, the study could not include a test–retest reliability analysis, which is essential for assessing the consistency of the scales over time. Additionally, criterion validity analysis was not performed because a suitable scale for comparison was not available, highlighting a gap that future research should address. Despite these limitations, the study provides a valuable foundation for further exploration of maternal gatekeeping in infancy.

## 5 Conclusion

**Figure 3:**
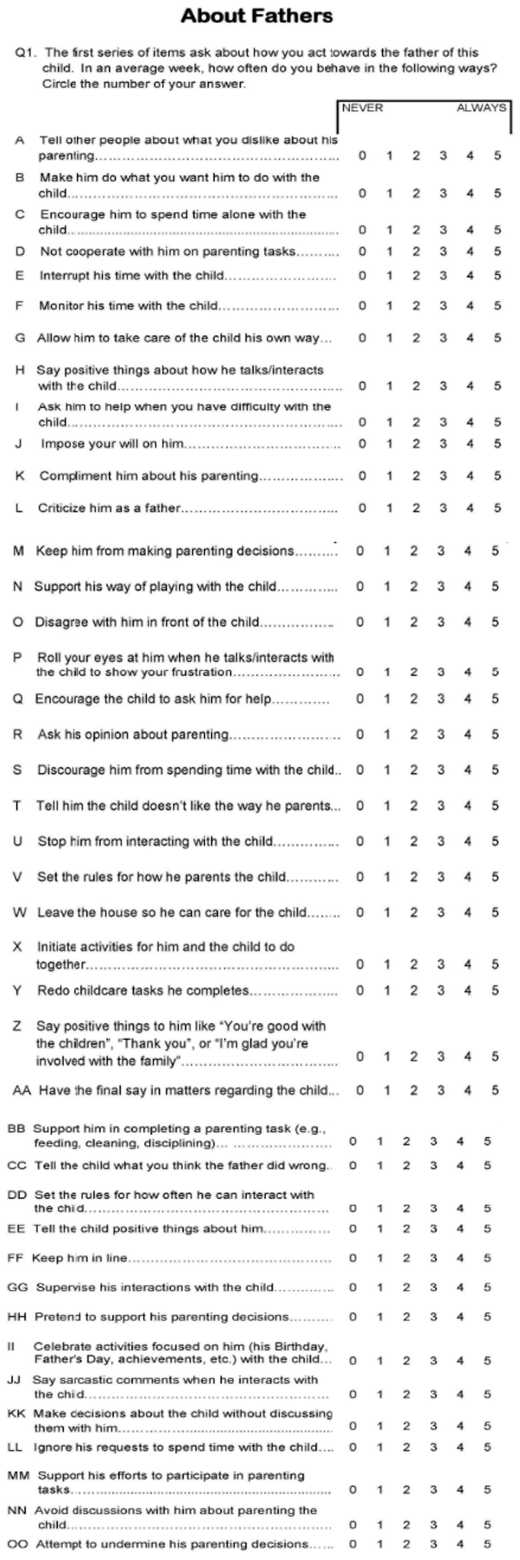
English (original) version of Maternal Gatekeeping Scale.

**Figure 4:**
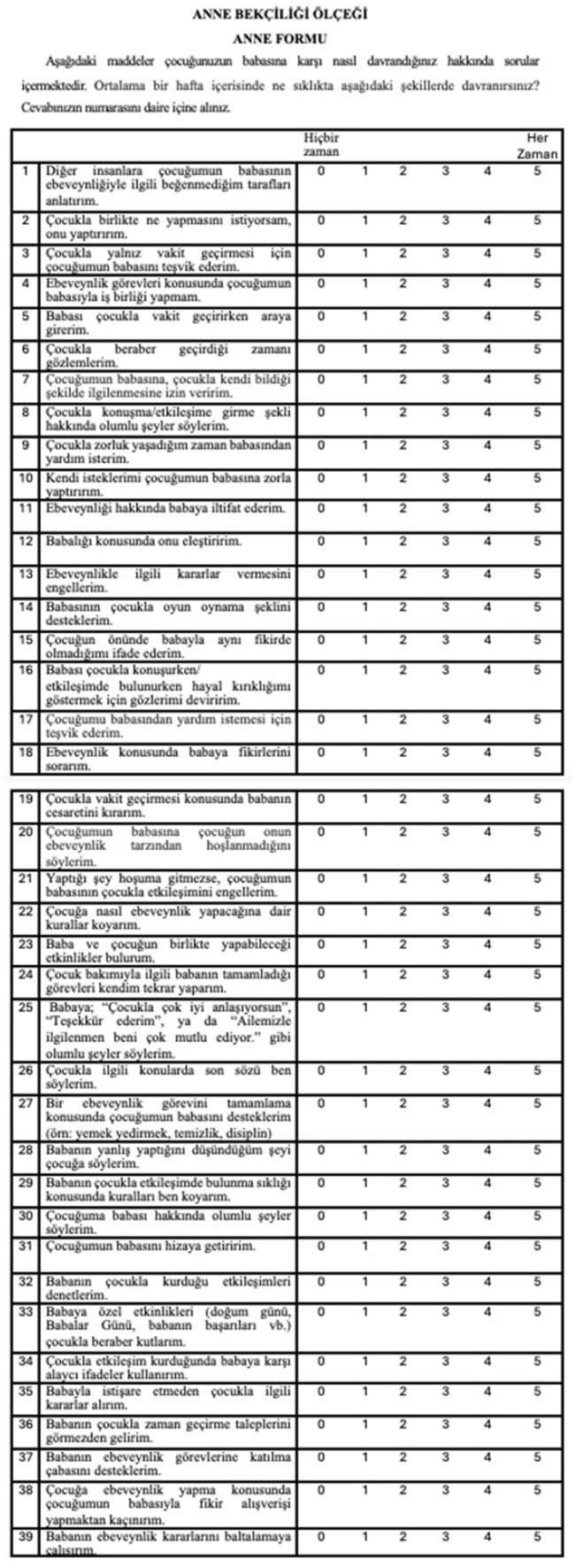
Turkish version of the scale Maternal Gatekeeping Scale- Mother Form for infancy period (0–24 Months).

**Figure 5:**
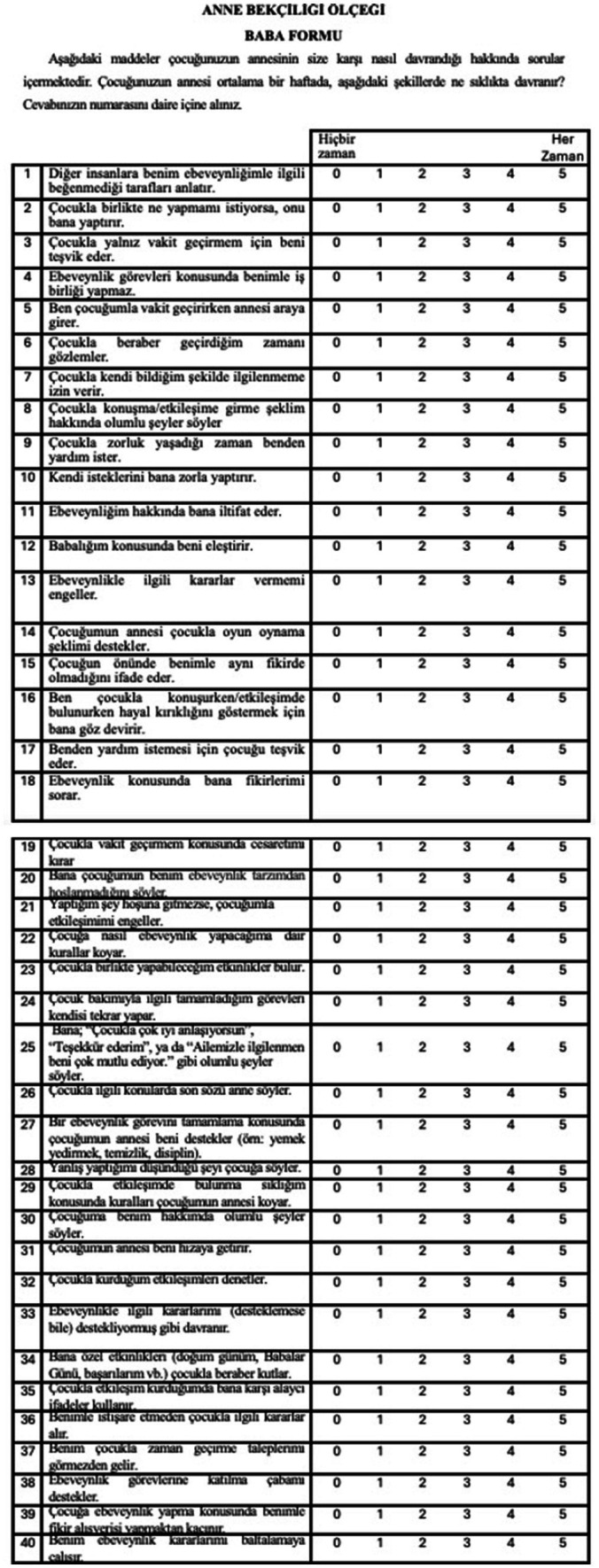
Turkish version of the scale Maternal Gatekeeping Scale- Father Form for infancy period (0–24 Months).

In this study, the Maternal Gatekeeping Scale was adapted into Turkish for use in infancy and validity and reliability studies were conducted with parents. Thus, Maternal Gatekeeping Scale-Mother Form and Maternal Gatekeeping Scale-Father Form were obtained for infancy. The study examined the validity and reliability of the Maternal Gatekeeping Scale S based on data collected from married and cohabiting parents whose children were in infancy (0–24 months). The results of the study show that the Maternal Gatekeeping Scale is a valid and reliable instrument for measuring maternal gatekeeping by collecting data from mothers and fathers in Türkiye. English version of the scale is shown in Figure 3. Turkish versions of the scales is shown in Figures 4, 5.

## Data Availability

The raw data supporting the conclusions of this article will be made available by the authors, without undue reservation.
